# The Physiology of Man, Vol. V

**Published:** 1874-09

**Authors:** 


### Books Reviewed.

*The Physiology of Man; designed to represent the Existing state of Physiological Science as applied to the Functions of the Human Body.* By Austin Flint, Jr. M. D., etc. In Five Volumes—Vol. V. Special Senses; Generation. New York: D. Appleton & Co., 1874. Buffalo: Herger & Ulbrich.

The fifth and last volume of Dr. Flint's great work upon Physiology places in the hands of the medical profession a complete system of physiology which is surpassed by no other work known upon the subject. The appearance of each volume has given additional evidence of Dr. Flint's ability as an observer and author.

He has placed the whole domain of physiological literature under levy to make his work complete; but a thorough student and experimenter himself, he has accepted but little as true which he has not personally investigated and proven.

The first chapter treats of the sense of touch, muscular sense and sensibility, the tactile corpuscles and the venereal sense. The next chapter is devoted to the olfactory apparatus. He gives briefly the physiological anatomy of the parts concerned, and discusses the relation between the sense of taste and of smell. He denies the statements of Magendie and Bernard, that the sense of smell does not reside alone in the olfactory nerves, and shows conclusively the error in Magendie's experiments.

Chapters III., IV., V., and VI., are devoted to the optic nerves, the anatomy of the eye, refraction, vision, the functions and mechanism of the iris, the muscles of the eyeball and eyelids, the lachrymal apparatus, etc.

Chapters VII., VIII., and IX., consider in an admirable manner the auditory nerves and the sense of hearing, including the physics of sounds &c., Chapter IX upon the functions of the various portions of the auditory apparatus is a very interesting and valuable section of the work. Gustation is the topic of Chapter X. The physiological anatomy of the parts upon which this sense depends is considered in detail; the relation of gustation to the sense of smell, to facial paralysis and to paralysis of the tongue also occupies a portion of this chapter. The remaining chapters of the book, XI. to XIX., are devoted to the subject of generation and the development of the fœtus. Chapters XI and XII treat of the female organs of generation, the ovum and ovulation, puberty, menstruation, changes in the pregnant uterus, the corpus luteum &c. The researches of Dalton upon the corpus luteum of menstruation and pregnancy are referred to in complimentary terms. These investigations by Prof. Dalton, were made many of them, we believe in this city, and we have seen some of the original drawings made by him.

Chapter XIII gives a full account of the male organs of generation and their changes from infancy to childhood. Chapter XIV., deals with fecundation and the part of the male and female in the reproductive act. The author places some confidence in the views of Dr. Beck, of Fort Wayne, Indiana, as enunciated in the *St. Louis Medical and Surgical Joarnal*, vol. IX., page 449, and more recently in an article read before the American Medical Association at Detroit, in June, 1874. Dr. Beck asserts that during coitus, or a sexual orgasm the action of the uterus is similar to that in a case observed by him which he was treating for uterine disease. He says: during the orgasm "the os opened to the extent of fully an inch, made five or six successive gasps, drawing the external os into the cervix each time powerfully, and at the same time becoming quite soft to the touch." These observations have been confirmed by Litzmann and other writers. We had the pleasure of hearing Dr. Becks paper at Detroit, and see no reason for doubting the accuracy of his observation, yet we cannot accept this as the method of extrance of the spematozoids in all instances, as well authenticated cases are on record of impregnation where there was no orgasm on the part of the female and in which penetration even was not fully accomplished. We agree with the author however as to the value of Dr. Beck's paper, and that it has not received that attention which it deserves.

Prof. Flint does not think that any time may be designated in which impregnation would be impossible, the greatest amount of evidence, however, showing the time in which it is most apt to occur is that immediately following menstruation.

Chapter XV., XVI., XVII., and XVIII., treat of the developement of the embryo from the segmentation of the vitelleus to the time of birth.

Chapter XIX closes the work. It views the subjects of the duration of pregnancy, fœtal life, multiple pregnancy, the cause of the first contractions of the uterus in normal parturition, involution of the uterus, meconium dextral pre-eminence, developement after birth, age, death, cadaveric rigidity and putrefaction.

Thus concludes a work upon phys'ology which has no superiors, few equals. Written in a country where the author labored under the disadvantages of having no large and well regulated libraries to consult, it evinces a wide range of reading in every department of physiological science and in all languages.

The mechanical execution of the work is a credit to Messrs. Appleton & Co., the paper is clear and smooth, and the type large and plain. No physician's library is complete without this, the best Treatise upon Human Physiology.

